# The implications of hyperoxia, type 1 diabetes and sex on cardiovascular physiology in mice

**DOI:** 10.1038/s41598-021-02550-2

**Published:** 2021-11-29

**Authors:** Katarina Bojkovic, Jennifer Leigh Rodgers, Riddhi Vichare, Asmita Nandi, Noah Mansour, Faizan Saleem, Zain Ul Abidin, Sahit Vanthenapalli, Feng Cheng, Siva Kumar Panguluri

**Affiliations:** Department of Pharmaceutical Sciences, College of Pharmacy, University of South Florida, 12901 Bruce B. Downs Blvd., MDC-30, Tampa, FL 33612 USA

**Keywords:** Cardiovascular biology, Molecular medicine

## Abstract

Oxygen supplementation, although a cornerstone of emergency and cardiovascular medicine, often results in hyperoxia, a condition characterized by excessive tissue oxygen which results in adverse cardiac remodeling and subsequent injurious effects to physiological function. Cardiac remodeling is further influenced by various risk factors, including pre-existing conditions and sex. Thus, the purpose of this experiment was to investigate cardiac remodeling in Type I Diabetic (Akita) mice subjected to hyperoxic treatment. Overall, we demonstrated that Akita mice experience distinct challenges from wild type (WT) mice. Specifically, Akita males at both normoxia and hyperoxia showed significant decreases in body and heart weights, prolonged PR, QRS, and QTc intervals, and reduced %EF and %FS at normoxia compared to WT controls. Moreover, Akita males largely resemble female mice (both WT and Akita) with regards to the parameters studied. Finally, statistical analysis revealed hyperoxia to have the greatest influence on cardiac pathophysiology, followed by sex, and finally genotype. Taken together, our data suggest that Type I diabetic patients may have distinct cardiac pathophysiology under hyperoxia compared to uncomplicated patients, with males being at high risk. These findings can be used to enhance provision of care in ICU patients with Type I diabetes as a comorbid condition.

## Introduction

Modulations to cardiac physiology often occur after oxygen supplementation, an integral component of cardiovascular medicine used in the intensive care unit (ICU) to treat hypoxia, or low oxygen levels in bodily tissues^1,2^. Unfortunately, this treatment may also result in hyperoxia, a condition where excessive tissue oxygen culminates in injurious physiological effects, such as cellular death, increased presence of reactive oxygen species (ROS), and overall increased mortality compared to normoxic patients^2,3^. Importantly, oxygen supplementation is the most commonly used treatment administered to COVID-19 patients^4^, and although the effects of hyperoxic exposure have not been studied in this patient population specifically, it is well established that oxygen toxicity negatively impacts physiological and cardiovascular function. Thus, it is imperative to consider the risk factors that will influence cardiac remodeling in the ICU.

Individuals with existing comorbidities are more likely to be admitted to the ICU than those without such conditions, and they are more likely to experience hyperoxic treatment and subsequent cardiac remodeling as a result^5–7^. Importantly, diabetes mellitus (DM) patients comprise anywhere from 9.3–25% of the ICU patient population^8,9^. Moreover, there are well-established distinctions between male and female respiratory function in both the animal and clinical models^10,11^. Thus, this experiment aims to investigate cardiac remodeling in normoxic and hyperoxic male and female T1DM mice to determine the intersections between T1DM, hyperoxic treatment, and sex on cardiovascular physiology.

In our studies, we have shown adult male wild‐type (WT) mice subjected to 72 h hyperoxic conditions display clear cardiac pathophysiology, including bradycardia, redox abnormalities, QTc and JT prolongation, dysregulation of ion channels, and elevated levels of serum markers of cardiac injury^12–14^. In the clinical model, hyperoxia is associated with vasoconstriction, apoptosis, pulmonary damage, oxidative stress, and death^15–17^.

Such outcomes are further influenced by gender-dependent risk factors, such as male and female hormones. For example, estrogen decreases the incidence and prevalence of cardiovascular disease (CVD) in women compared to age-matched men^18,19^. Still, while it is accepted that the ovarian hormone estrogen confers cardioprotection in women, studies have shown that testosterone may exert cardioprotective effects in men^20^. In spite of this, testosterone is also associated with cardiac hypertrophy and adverse cardiac events, such as myocardial infarction^21^. Thus, although hormonal fluctuations are implicated in cardiac structure and function, there is no consensus as to how to achieve optimal levels for cardioprotection.

Our previous research has shown that hyperoxia induces cardiac remodeling at the physical, functional, electrophysiological, biochemical, and molecular levels in the murine model^7^. Moreover, we have shown that the aforementioned outcomes differ between hyperoxic male and female mice^14^, as well as Type-2 diabetic mice compared to WT mice^22^. However, the effects of hyperoxia in T1DM mice have not been the focus of any major study thus far. Therefore, this pioneer study investigates the intersection between the following three risk factors which influence cardiovascular physiology: hyperoxic treatment, T1DM, and gender. Finally, Multi-Way Analysis of Variance (ANOVA) at completion of the experiment calculated the statistical influence of each risk factor on the study parameters, while the Pearson's correlation matrix of outcomes determined the statistical correlation between them. Through this study, we aim to inform the treatment of male and female Type-1 Diabetic patients in the ICU or emergency care, specifically as it pertains to emergent supplemental oxygen.

## Results

### Physical parameters

**Figure 1:**
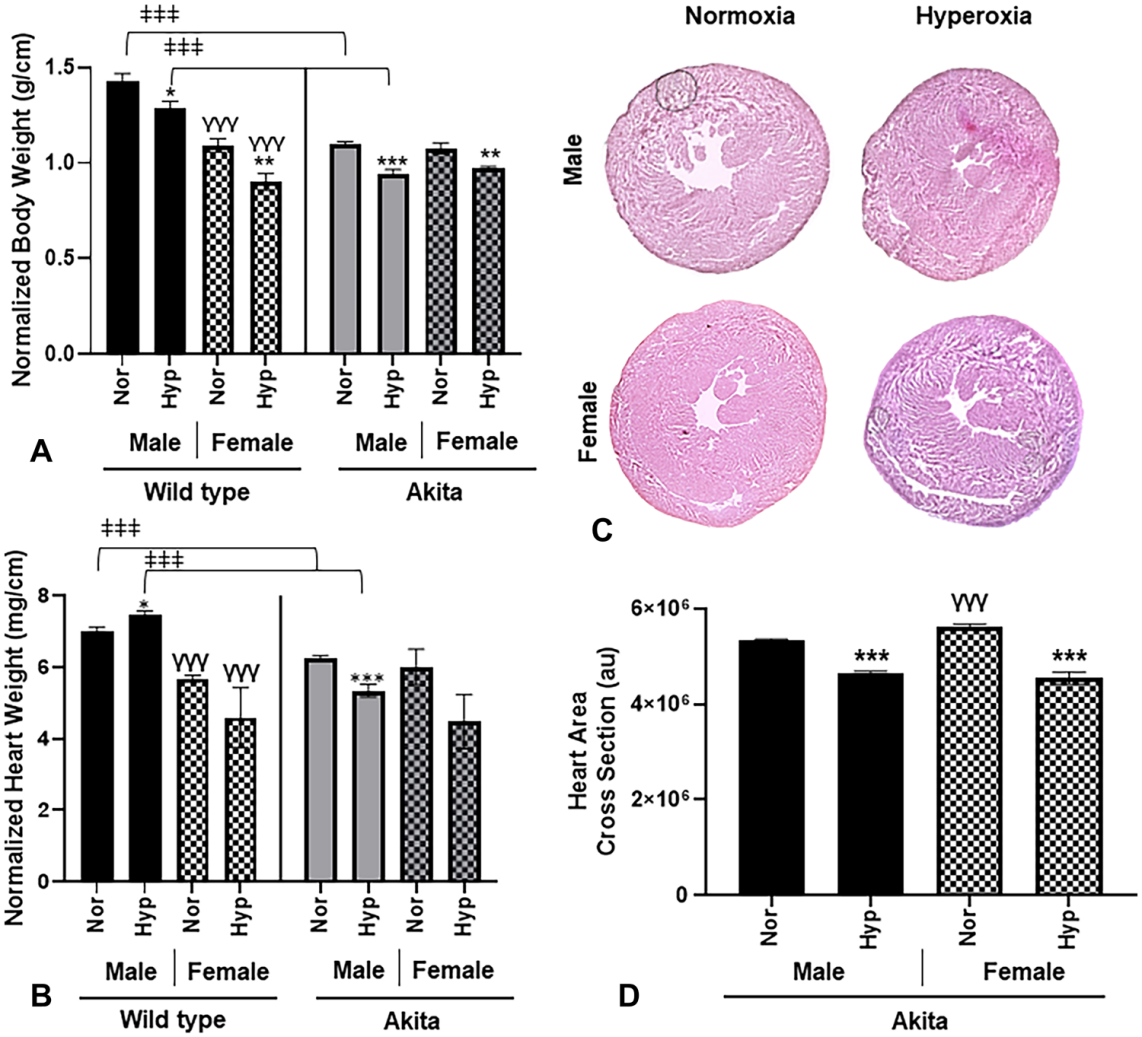

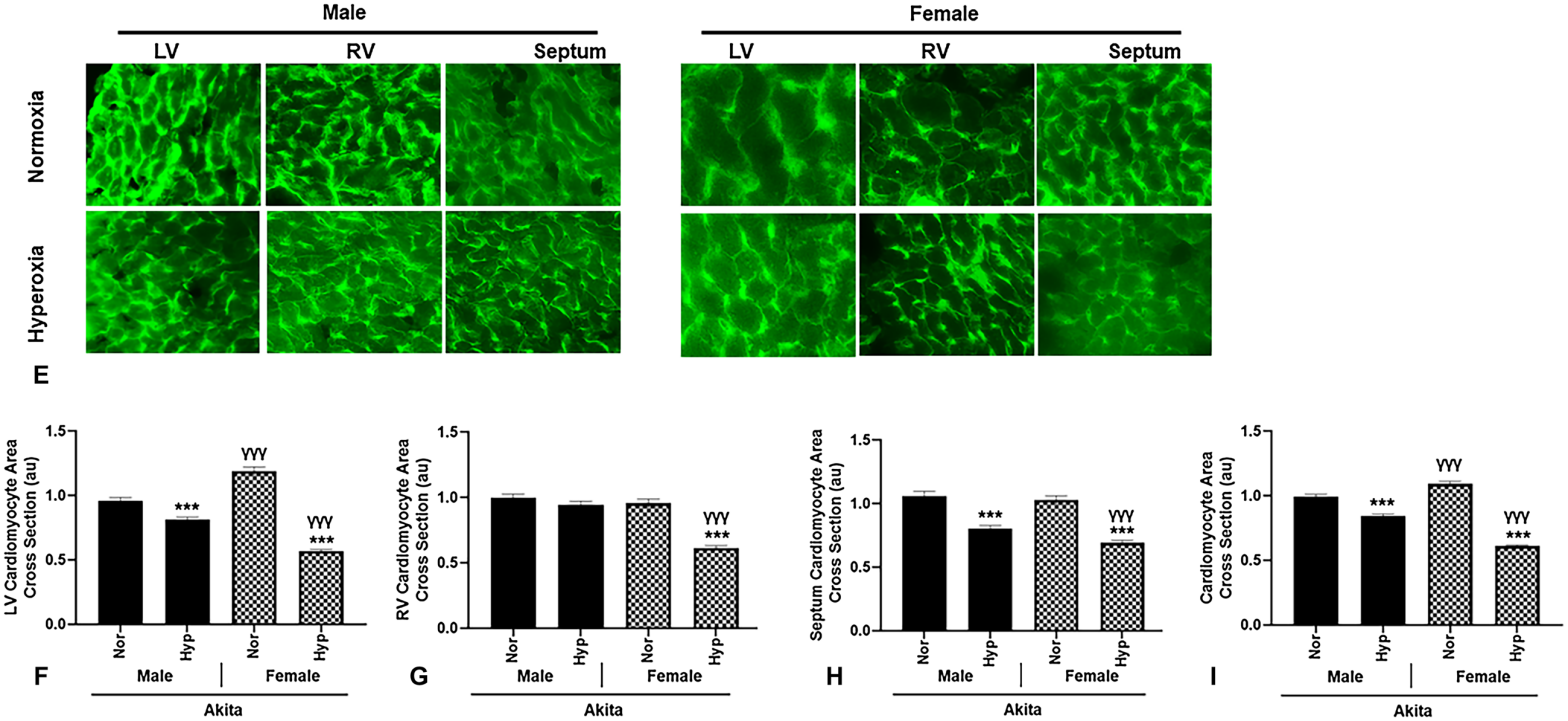
Akita male mice at both normoxia and hyperoxia experience a significant decrease in body and heart weight. (**A**) Body weight normalized to tibia length (g/cm) for all experimental groups, (**B**) Heart weight normalized to tibia length for all experimental groups (mg/cm), (**C**) H&E histological cross sections of Akita male and female mice under normoxia and hyperoxia, (**D**) Heart area cross sections (au), (**E**) WGA stained cardiac myocytes stained from Akita male and female mice. (**F**–**I**) Cardiac myocyte area of Akita male and female mice under normoxia or hyperoxia shown as follows: (**F**) LV cardiomyocte area, (**G**) RV cardiomyocte area, (**H**) Septum cardiomyocte area, (**I**) Pooled cardiomyocte area. *Error bars represent* ± SEM. *p < 0.05, **p < 0.005 ***p < 0.0005. *compares effects of hyperoxia and normoxia of same gender and strain; Y compares male and female mice of the same strain and treatment; ‡compares WT and Akita of same gender and treatment.

As we have previously demonstrated hyperoxia significantly reduces body weight in mice^13,14,22^, we compared body weights of Akita mice before and after normoxic and hyperoxic treatment. As expected, all mice exposed to hyperoxia experienced a significant decrease in body weight compared to normoxic controls (Fig. 1A). Regarding Akita mice, males at both baseline normoxia and hyperoxia experienced a significant (*p* < 0.0005) decrease in body weight compared to WT male mice exposed to either treatment (Fig. 1A).

In addition to body weight, we also examined heart weights, finding that Akita male heart weights were significantly decreased (*p* < 0.0005) at both normoxia and hyperoxia compared to WT males (Fig. 1B). Additionally, while WT male mice experienced a significant increase (*p* < 0.05) in heart weight after hyperoxic treatment, Akita male mice experienced a significant decrease in this parameter (*p* < 0.0005).

After observing significant changes in heart weights under hyperoxia, we elected to measure heart cross sectional areas. We found significantly smaller (*p* < 0.0005) heart cross sectional areas in all hyperoxic Akita mice compared to normoxia controls (Fig. 1C, D). We then measured cardiomyocyte area, cardiomyocyte area in hyperoxic mice from LV, RV (except Akita male), septum, and in the pooled average showed significantly smaller (*p* < 0.0005) than normoxia correlates (Fig. 1E–I). Although Akita females exhibited significantly larger cardiomyocytes than Akita males at normal air, these cells became significantly smaller than those of Akita males after hyperoxia.

### Functional parameters

After observation of significant changes in physical parameters post hyperoxic treatment in the experimental mice, we placed the animals under mild anesthesia for echocardiogram (echo) analysis to determine possible modifications to cardiac functional parameters.

**Figure 2:**
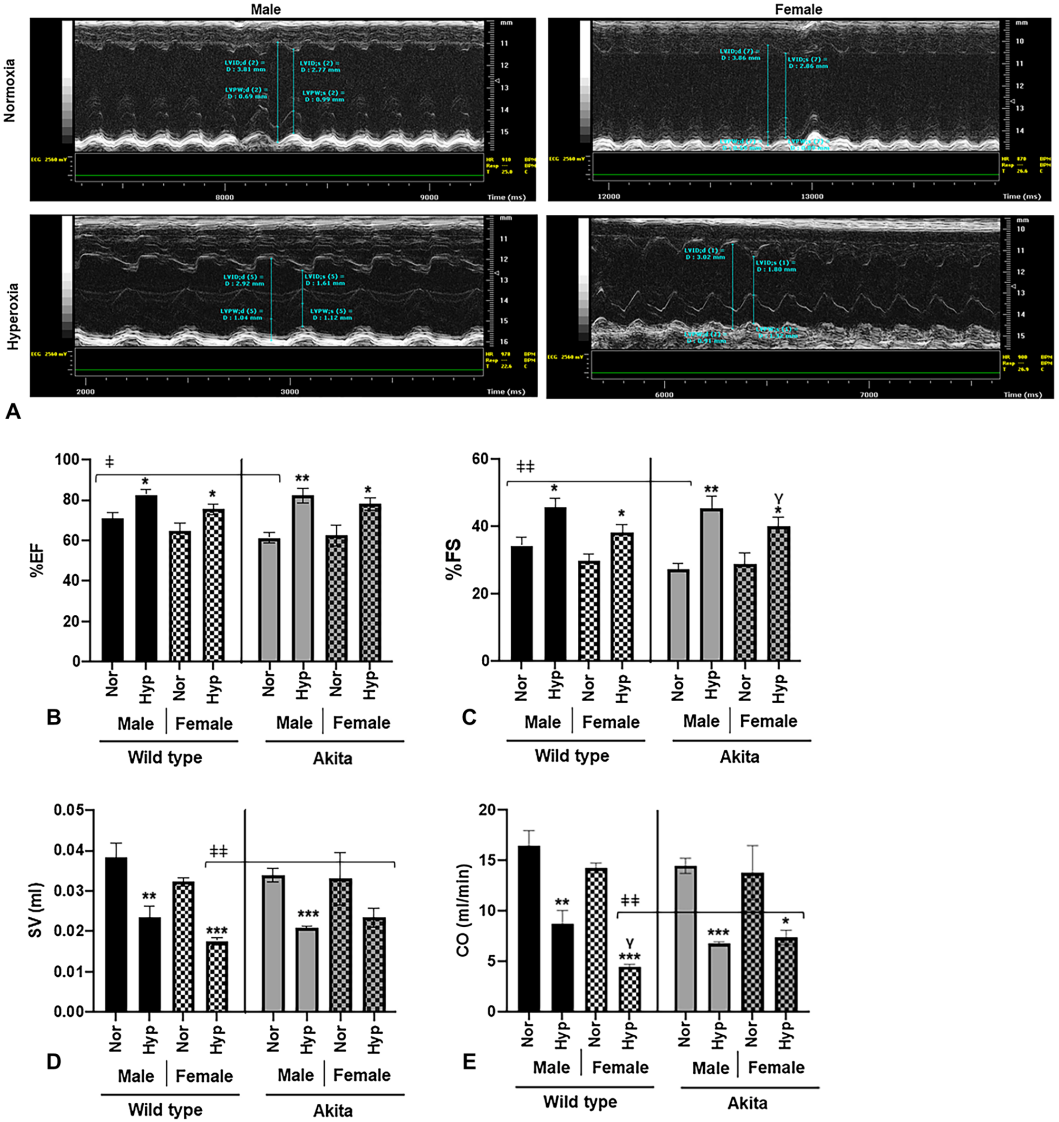
Akita mice display distinct functional parameters compared to WT mice (**A**) percent ejection fraction (FS), (**B**) percent fractional shortening (EF), (**C**) stroke volume (SV), and (**D**) cardiac output (CO) in hyperoxia/normoxia treated Akita and wild type mice, (**E**) Optical traces for male and female Akita mice in normoxic and hyperoxic conditions*. Error bars represent* ± SEM. *p < 0.05, **p < 0.005 ***p < 0.0005. *compares effects of hyperoxia and normoxia of same gender and strain; Y compares male and female mice of the same strain and treatment; ‡compares WT and Akita of same gender and treatment.

Left ventricular internal diameter end-diastolic and end-systolic (LVIDd and LVIDs, respectively) measurements significantly decreased in all hyperoxic mice compared to normoxia correlates (Fig. 2A and Table 1). Consequently, end-diastolic volume and end-systolic volume (EDV and ESV, respectively) significantly decreased after hyperoxia as well (Table 1). We also report that hyperoxic treatment significantly increased % ejection fraction (EF) and % fractional shortening (FS) in all mice (Fig. 2B, C). Additionally, cardiac output (CO) and stroke volume (SV) were significantly decreased in all mice after hyperoxic treatment (Fig. 2D, E).

**Table 1:** Echocardiogram parameters after hyperoxia treatment.

	LVIDd	LVIDs	EDV	ESV
**WT male**				
Normoxia	3.76 (± 0.11)	2.48 (± 0.12)	54.2 (± 4.61)	15.9 (± 2.12)
Hyperoxia	3.01 (± 0.16)***	1.66 (± 0.16)***	29.4 (± 4.26)***	6.01(± 1.83)***
**WT female**				
Normoxia	3.7 (± 0.12)	2.61 (± 0.18)	51.1 (± 5.28)	18.9 (± 4.36)
Hyperoxia	2.86 (± 0.08)***	1.78 (± 0.11)***	23.7 (± 1.80)***	6.12 (± 0.97)***
**Akita male**				
Normoxia	3.82 (± 0.09)	2.78 (± 0.12)	56.0 (± 3.91)	22.1 (± 2.89)
Hyperoxia	2.94 (± 0.04)***	1.62 (± .0.13)***	25.6 (± 1.15)***	4.66 (± 1.17)***
**Akita female**				
Normoxia	3.70 (± 0.19)	2.64 (± 0.18)	52.8 (± 8.12)	19.6 (± 3.19)
Hyperoxia	3.1 (± 0.13)**	1.86 (± 0.15)***	30.6 (± 3.78)**	7.11 (± 1.78)***

Values presented here are mean (n = 6 to 10 ± *SEM*).

**represents p < 0.005 and ***p < 0.0005.

However, some functional parameters observed in Akita mice deviate from those of WT mice at both normoxia and hyperoxia. Namely, at baseline normoxia, Akita males demonstrate a significant reduction in both %EF and %FS (*p* < 0.05 and *p* < 0.005, respectively) compared to WT correlates (Fig. 2B, C). Additionally, when comparing females between strains at hyperoxia, SV and CO modulations in Akita females were worsened to a significantly lesser extent (*p* < 0.005) compared to WT females (Fig. 2D, E).

### Electrophysiological parameters

**Figure 3:**
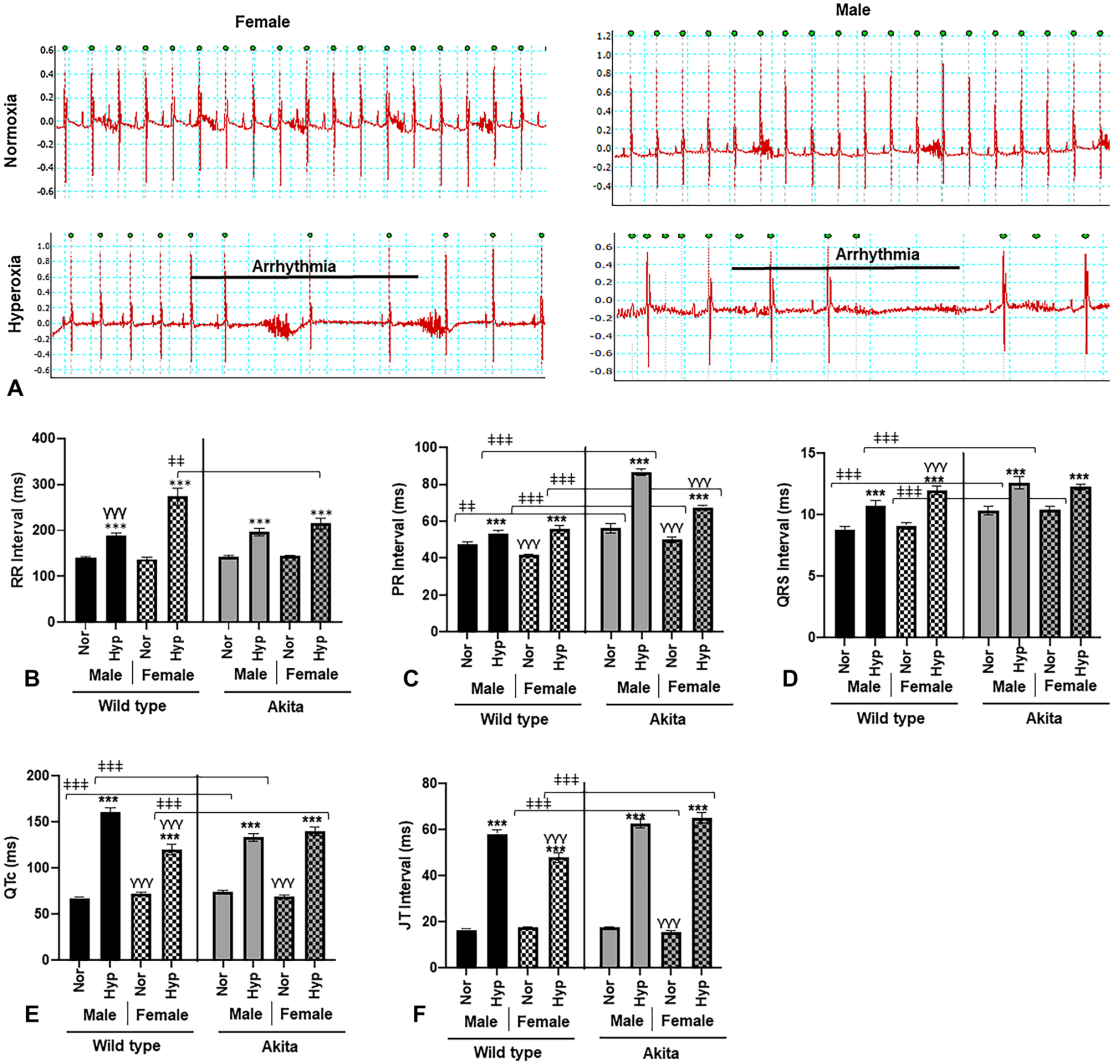
Hyperoxia induces bradyarrhythmias in Akita (and WT) mice (**A**) Original ECG traces in lead II mode in normoxia/hyperoxia treated Akita male and female mice, (**B**) RR interval, (**C**) PR interval, (**D**) QRS interval, (**E**) QTc interval, (**F**) JT interval. *Error bars represent* ± SEM. *p < 0.05, **p < 0.005 ***p < 0.0005. *compares effects of hyperoxia and normoxia of same gender and strain; Y compares male and female mice of the same strain and treatment; ‡compares WT and Akita of same gender and treatment.

After finding hyperoxia significantly altered functional parameters, we elected to conduct surface ECG to investigate electrophysiological modulations. Compared to normoxia treatment, hyperoxic treatment significantly increased (*p* < 0.0005) all parameters observed, regardless of gender or strain (Fig. 3B–F). Of note, we observed brady-arrhythmias in both male and female Akita mice (Fig. 3A, B), similar to what we previously observed in WT mice^14,22,23^.

Pertaining to Akita mice, Akita males demonstrated distinct features in electrophysiology compared to WT males at both normoxia and hyperoxia. At normoxia, Akita male mice experience significant increases in PR, QRS, and QTc intervals compared to their WT counterparts (Fig. 3C–E). Interestingly, the QTc interval in normoxic Akita male mice is significantly larger than that of Akita females at normal air, which is distinct from what we reported in WT controls^14^. At hyperoxia, Akita males experienced increased PR and QRS intervals, and while all mice experienced elevated QTc after hyperoxia, the QTc prolongation of hyperoxic Akita males was much more similar to the QTc prolongation observed in hyperoxic female mice from either strain than that experienced by hyperoxic WT males.

### Biochemical markers

**Figure 4:**
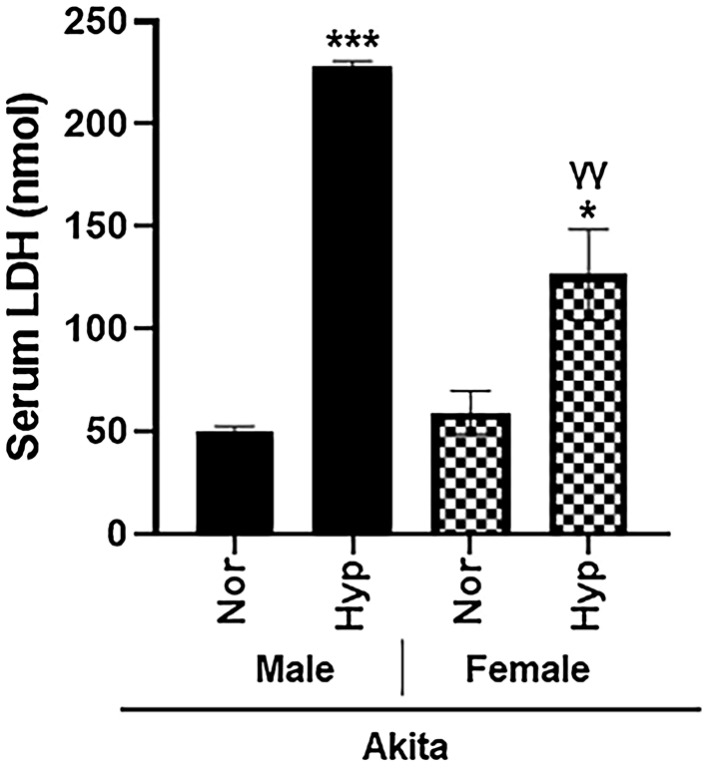
Hyperoxia induce cardiotoxicity in Akita mice as evident by elevated levels of serum LDH in both sexes. Lactic Dehydrogenase (LDH) measured in serum from normoxia and hyperoxia WT and Akita groups. *Error bars represent* ± SEM. *p < 0.05, **p < 0.005 ***p < 0.0005. *compares effects of hyperoxia and normoxia of same gender and strain; Y compares male and female mice of the same strain and treatment.

A biochemical assay was performed after hyperoxic treatment to evaluate serum levels of lactate dehydrogenase (LDH), a well-established biomarker of cardiac injury. As we have reported in hyperoxic WT mice^13,22^, hyperoxic Akita mice also demonstrated a significant increase in serum LDH (Fig. 4). Of note, hyperoxic Akita males experienced a significantly higher (*p* < 0.0005) increase in serum LDH levels than hyperoxic Akita females (*p* < 0.05).

**Figure 5:**
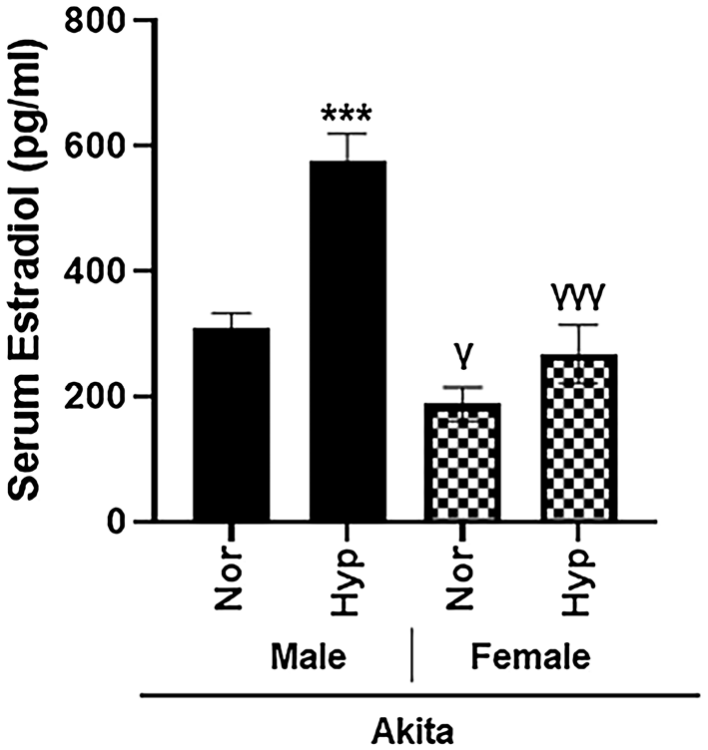
Dysregulation of serum estradiol in Akita male mice in both normal air and hyperoxia. Estradiol levels were measured in serum from normoxia and hyperoxia WT and Akita groups using ELISA kit. *Error bars represent* ± SEM. *p < 0.05, **p < 0.005 ***p < 0.0005. *compares effects of hyperoxia and normoxia of same gender and strain; Y compares male and female mice of the same strain and treatment.

Additionally, due to the significantly smaller size of Akita male mice, we assessed serum estradiol levels to determine potential hormonal fluctuations between strains. Akita males at both normoxia and hyperoxia demonstrated significantly higher serum estradiol levels (*p* < 0.05 and *p* < 0.005, respectively) than Akita females exposed to either treatment (Fig. 5).

### Statistical analysis

To determine the statistical influence of the three risk factors (hyperoxia, T1DM, and sex), Multi-Way Analysis of Variance (ANOVA) was performed. From our data, genotype was found to have the greatest influence in terms of statistical significance for physical parameters, whereas hyperoxia exhibited the greatest effect on functional and electrophysiological parameters, followed by genotype and sex (Table 2). The Pearson correlation coefficient was also calculated for these parameters, demonstrating in almost all groups a positive correlation between heart and body weights, but a negative correlation between heart rates and QRS and/or JT intervals (see Supplemental material).

**Table 2:** Multi-way analysis of variance (ANOVA).

	Genotype		Sex		Treatment	
	Pr(> F)	Adjusted *p*-value	Pr(> F)	Adjusted *p*-value	Pr(> F)	Adjusted *p*-value
Body weight	1.71E−08	0	2.76E−06	3.90E−06	0.000218	0.0002209
Heart weight	1.16E−06	1.20E−06	1.44E−06	2.00E−06	0.267	2.67E−01
Heart area	< 2E−16	0	4.24E−09	0	0.276	0.2829724
%EF	0.0851	0.0850554	0.0665	0.0673891	1.59E−07	2.00E−07
%FS	0.1248	0.1248431	0.0358	0.0363352	1.87E−07	2.00E−07
CO	0.3346	0.3346175	0.0432	0.043897	6.20E−13	0
SV	0.597	0.5974075	0.169	0.1707943	1.16E−07	1.00E−07
LVID;s	0.142	0.1415086	0.529	0.5299796	6.93E−11	0
LVID;s	0.0702	0.0701931	0.3876	0.3891139	3.27E−11	0
RR	7.38E−08	1.00E−07	0.0223	0.0235337	< 2.00E−16	0.00E+00
PR	< 2.00E−16	0	1.37E−05	1.63E−05	< 2.00E−16	0
QRS	7.44E−07	7.00E−07	0.21	0.2142415	4.66E−14	0
QTC	0.08964	0.0896405	0.00039	0.0004383	< 2.00E−16	0.00E+00
JT	0.0017	0.0017007	0.0171	0.0181318	< 2E−16	0

## Discussion

Oxygen supplementation, though an indispensable mechanism of treatment in the ICU, negatively impacts cardiovascular parameters and increases mortality rates compared to normoxia treatment^24,25^. A retrospective cohort study of ninety-seven ICU facilities found that the percentage of patients receiving supplemental oxygen therapy at any hour was 39.5% (± 15.2%)^5^, indicating that many ICU patients will receive oxygen supplementation at some point and, in turn, become exposed to hyperoxic conditions. Moreover, the use of emergent supplemental oxygen is increasing on a global scale, as it is the most commonly used treatment for COVID-19 patients^4^. To reiterate, men and women exhibit inherent differences in respiratory function. Moreover, those that present most frequently to the ICU are individuals suffering from existing comorbidities, and one of the most common of such conditions is diabetes mellitus. Thus, it is imperative to understand how cardiac remodeling is influenced by hyperoxia, T1DM, and sex.

In line with our previous findings^14,26,27^, we found all mice exposed to hyperoxic conditions, irrespective of gender or strain, experienced a significant decrease in body weight compared to normoxia controls (Fig. 1A). We also observed that Akita male mice at both baseline normoxia and hyperoxia experienced a significant (*p* < 0.0005) decrease in body weight compared to WT male mice exposed to either treatment (Fig. 1A).

With respect to heart weight, we have previously established that hyperoxia increases heart weights in WT males, while heart weights of WT females decrease^14,22,23^. Interestingly, we found that Akita males experience a decrease in heart weight after hyperoxia, similar to what is observed in hyperoxic female mice (both Akita and WT) (Fig. 1B). Moreover, Akita LV, RV, septum, and pooled cardiomyocyte areas significantly decreased after hyperoxia (with the exception of Akita male RV cardiomyocytes, which did decrease albeit not in a statistically significant manner) (Fig. 1E–I).

Regarding hormonal fluctuations, Akita males exhibit significantly increased (*p* < 0.05) serum estradiol levels compared to Akita females (Fig. 5). Moreover, while studies have reported significantly decreased androgen levels in Akita males^28,29^, we are the first to report increased estradiol levels in male Akita mice compared to their female counterparts at both baseline normoxia and hyperoxia conditions.

It is important to address that the Akita phenotype, which includes hyperglycemia, hypoinsulinemia, polydipsia, and polyuria, is more exaggerated in male mice compared to females^30^. Because hyperglycemia is a well-studied risk factor for cardiovascular disease and mortality^31^, resulting in both micro and macrovascular complications that induce cardiovascular stress, this metabolic process may further exacerbate cardiovascular pathophysiology in male mice compared to females overall.

Concerning the small size of Akita male mice, the T1DM genotype may be influential. In the clinical model, T1DM is associated with sub-optimal growth due to microvascular complications such as nephropathy^32^. Additionally, the decreased serum levels of testosterone which have been previously observed in Akita males also likely contributes to their smaller size (44, 45). Testosterone has an integral role in glucose-stimulated insulin secretion (GSIS) in men^33^, and recent reports indicate testosterone deficiency is common in men with both T1DM and T2DM^33–35^. Thus, testosterone deficiency as a consequence of the T1DM genotype may lead to impaired insulin secretion, thereby making weight gain more difficult.

The T1DM genotype may also contribute to the observation that hyperoxic Akita male mice did not display cardiac hypertrophy but instead experienced reductions in heart size similar to female mice. In cardiomyocytes, testosterone induces hypertrophy through both activation of the rapamycin complex 1 (mTORC1) pathway and glucose uptake facilitated by AMP-activated protein kinase (AMPK) activation^36^. Moreover, androgen receptors themselves can alter the cardiac phenotype by modulating hypertrophy in cardiac myocytes^37,38^. Taken together, these factors may help explain why Akita male mice do not experience hyperoxia-induced cardiac hypertrophy, which was otherwise evident in wild-type male mice as we reported previously^14,23,39^.

Finally, statistical analysis determined genotype to have the most significant effect on physical parameters. Importantly, we also observed a positive correlation between heart and body weights, but a negative correlation between heart rates and QRS and/or JT intervals (Supplemental material). Studies have shown that in malnourished adults, body and heart weight decrease in tandem, while the resultant cardiac hypotrophy is associated with a prolonged QTc interval^40,41^, as observed in this experiment. Thus, the T1DM genotype has an important role in influencing physical parameters in Akita male mice, which we believe influence electrophysiological outcomes. Moreover, this influence is evident at both baseline normoxia and hyperoxia.

In the clinical setting, hyperoxic treatment results in modulations to functional parameters such as decreased cardiac output (CO) and stroke volume (SV)^42,43^. Importantly, studies have shown T1DM patients may experience various functional parameter abnormalities even at normoxia, such as significantly lower %EF^44^. Thus, hyperoxic treatment in T1DM patients may augment existing cardiac pathophysiology in this population.

Previously, we have reported that LVIDd, LVIDs, ESV, and EDV significantly decrease after hyperoxia in WT mice^14,23^. Additionally, we also reported CO and SV in WT mice along with hyper-dynamic left ventricular ejection fraction (HDLVEF)^13,14^. Our data from this experiment correspond well to these findings in that all mice, irrespective of gender or strain, experienced significant decreases in LVIDd, LVIDs, ESV, EDV, SV and CO (Table 1 and Fig. 2D, E), in addition to a significant increase in %FS and %EF compared to normoxia counterparts (Fig. 2B, C).

Abnormal electrocardiographic readings of T1DM patients are relatively common (about 35% at baseline)^45^, increasing in number and severity over time^46^. In this experiment, all experimental mice demonstrated bradycardia under hyperoxia, as demonstrated by the significant increases in RR intervals compared to normoxia controls (Fig. 3B). While T1DM is not independently associated with bradycardia, such arrhythmias have been observed in hypoglycemic events^47^, an important consideration for administration of supplemental oxygen to this patient population.

With regards to Akita mice specifically, atrioventricular conduction abnormalities were observed even at normal air and were further augmented by hyperoxic treatment, signified by the increased PR intervals (Fig. 3C). Importantly, the prolonged PR interval is associated with increased risk of atrial fibrillation (AF), as well as increased risk of mortality^48^. Additionally, we observed ventricular depolarization abnormalities even at normal air in Akita mice, which were further augmented by hyperoxic treatment, as signified by the increased QRS intervals (Fig. 3D). Interestingly, Akita male mice also demonstrated elevated QTc intervals compared to female mice from either strain, indicating ventricular repolarization defects in Akita males. (Fig. 3E). Although it is widely accepted that females have higher QTc intervals than males, a prolonged QTc interval is a relatively common finding in T1DM patients overall^49,50^. The prolongation of the QTc interval is independently associated with hypoglycemic attacks in T1DM patients and is an independent marker of mortality within this patient population^51,52^. Therefore, male T1DM patients may be at greater risk for these adverse outcomes, and the hyperoxia-induced increase in QTc prolongation should therefore be considered as an additional risk factor when treating this subgroup. Moreover, the significantly higher (*p* < 0.005) increase in serum LDH levels in Akita males after hyperoxia treatment compared to hyperoxic Akita females (Fig. 4) further elucidates such concerns.

To determine the mathematical impact of the risk factors studied (hyperoxia, gender, and T1DM genotype), Multi-Way Analysis of Variance (ANOVA) was performed. From our analysis, we determined physical parameters were influenced by all three risk factors, with genotype being the most statistically significant, followed by gender, and finally hyperoxia (Table 2). With regards to functional and electrophysiological parameters, hyperoxia had the greatest impact, followed by sex and genotype. Moreover, for almost all groups, correlation analysis indicated a positive correlation between body and heart weights, whereas a negative correlation was observed between heart rates and QRS and/or JT intervals (see Supplemental material), indicating possible influence of physical parameters on cardiac electrophysiology. Taken together, this data suggests that hyperoxic treatment has direct implications on cardiovascular function, which may augment inherent cardiac challenges experienced by T1DM patients.

## Conclusion

Emergent supplemental oxygen, although an integral component of supportive care in cardiovascular medicine and the ICU, is not without risk to physiological and cardiovascular function. Due to the high prevalence of patients with diabetes mellitus in the ICU that thereby may be subjected to hyperoxia treatment, we have previously elected to observe T2DM mice and found modulations to physical, functional, and electrophysiological parameters^22^. However, with this study, we are the first to investigate T1DM (Akita) mice under both normoxia and hyperoxia exposure and compare these findings to WT controls.

Common findings in this experiment observed in all experimental mice after hyperoxic exposure include significant increases in %EF and %FS, significant decreases in SV, CO, induced bradycardia, and significant increases in RR, PR, and QTc intervals compared to normoxia controls. However, it should be noted that we also observed marked differences between Akita mice and WT counterparts, both at baseline normoxia and hyperoxia. Even at normal air, Akita male mice experience significantly reduced body and heart weight compared to WT males, reflecting parameters observed in female mice overall. Moreover, Akita males face inherent cardiac disadvantages even at normoxia, such as atrioventricular conduction abnormalities, ventricular depolarization abnormalities, and higher QTc than females from either strain. Further, upon exposure to hyperoxic conditions, these conditions were augmented, suggesting higher cardiac risk for Akita males compared to female and wild type controls.

Finally, when comparing the risk factors studied (hyperoxia, sex, and genotype), ANOVA statistical analysis calculated that of all risk factors, hyperoxia is the most detrimental, followed by sex, and finally genotype. Correlation analysis also revealed possible influence of physical parameters on cardiac electrophysiology. Thus, our findings demonstrate that Akita male mice under hyperoxia are at the highest risk for adverse cardiac remodeling and subsequent negative physiological outcomes. The results of this study can help inform the treatment of both male and female T1DM patients in the ICU. Still, additional research should determine the role of androgen insensitivity within the Akita mice population and its effects on cardiac structure and function under hyperoxic conditions.

## Methods

### Animals

Adult male and female heterozygous Ins2^Akita^ (Akita; Strain# 003,548) mice, which develop insulin dependent diabetes (Type I diabetes), at 10 weeks of age were purchased from Jackson Laboratory (Bar Harbor, ME). Wild type (WT), age-matched controls (C57BL/6 strain: 000,664) were also utilized. To predict the number of animals to be used in each group, we used a sample size calculator (http://www.biomath.info/power/prt.htm) and calculated the sample size based on our experimental groups. Akita and WT mice were divided into four groups based on gender and treatment (n = 6–10): Akita/WT male normoxia; Akita/WT male hyperoxia; Akita/WT female normoxia; and Akita/WT female hyperoxia. Veterinary staff at USF Health were available to monitor these mice during treatment, and all experimental protocols used have been approved by the Institutional Animal Care and Use Committee (IACUC) at the University of South Florida (Tampa, FL), in accordance with the US National Institutes of Health and the study was carried out in compliance with the ARRIVE guidelines.

### Hyperoxia exposure

Mice were subjected to either FiO_2_ > 90% (hyperoxia) for 72 h in an airtight chamber (50 × 50 × 30 cm), or normal air (normoxia) following the experimental setup discussed in a previous publication^7,13,23^. To ensure that the mice were constantly subjected to hyperoxia, oxygen levels were monitored using an oxygen analyzer (Vascular Technology, Chelmsford, MA) attached to the chamber. Subsequently, mice were anesthetized with isoflurane for surface ECG recordings and euthanized with intraperitoneal injection (IP) of 50 mg/kg euthasol for thoracotomy. Immediately, blood was collected and centrifuged at 5500 rpm for 5 min for blood plasma collection, and hearts were excised and dissected to separate the atrium, apex, right and left ventricles, and septum.

### Echocardiogram

Transthoracic echocardiography was performed using Vevo770 Ultrasonograph (VisualSonics) equipped with a 30 MHz transducer. Cardiac function was measured in all mice using previously published procedures for echocardiographic measurements^23^. Briefly, the mice were immobilized with 1–1.5% isoflurane and body temperature was maintained at 37 °C. 2-D mode in parasternal long and short axis at the mid-papillary muscle level were imaged. From this parasternal short axis view, the 2-D guided M-mode across the anterior wall and posterior wall were recorded. LVAW (left ventricular anterior wall), LVID (left ventricular interior diameter) and LVPW (left ventricular posterior wall thickness) at systole and diastole were measured. %EF (ejection fraction) was calculated as (EDV-ESV)/EDV*100%, and %FS (fractional shortening) was calculated by (LVID;d − LVID;s)/LVID;d * 100%. Stroke volume (SV) was calculated from the difference between end-diastolic volume (EDV) and end-systolic volume (ESV), while cardiac output (CO) is the product of heart rate (HR) and SV.

### Electrocardiography (ECG)

Experimental subjects were exposed to 1–2% isoflurane, following surface probe insertion in lead II configuration based on the experimental protocol published in a previous publication^13^. Briefly, ECG data was recorded for both sexes in three separate intervals of 30 s duration each. Post recording of the ECG and heart rate signals, data was analyzed using PowerLab system and LabChart 7.2 software (AD instruments, UK). Assessment of parameters like RR, PR, QRS, and JT intervals, with QT intervals measured from the start of Q peak up to T baseline were performed as previously defined^7,13,14^. QTc (heart rate corrected) intervals were calculated based on Bazett’s formula (QTc = QT/RR1/2).

### Cardiac marker assay

Collected serum plasma from the hyperoxia and normoxia experimental groups was evaluated to measure lactate dehydrogenase (LDH) levels. The cardiac marker levels were analyzed using an LDH assay kit (Sigma-Aldrich, MO, USA), following the protocol described previously in our laboratory^13^.

### Serum estradiol assay

Serum estradiol levels were measured from all groups using estradiol 17-beta ELISA kit (Cat#ab108667, Abcam, MA, USA) using manufacturer’s protocol. ELISA was done with internal duplicates and animal replicates (n = 6) from each group. Final concentrations were calculated using standard curve, and mean values were expressed as pg/ml of estradiol.

### Histological analysis

Hearts from each experimental group were excised for staining with hematoxylin & eosin (H&E) and FITC-labelled wheat germ agglutinin (WGA). For H&E staining, heart sections (10 µm thick) were stained according to our previously published protocol^14^. For heart cross-sectional area, areas for all experimental groups were calculated using *ImageJ* software. The calculated areas are expressed as mean (± SEM) values in bar diagrams. For WGA staining, hearts were sectioned at 10 µm thickness and fixed in 4% paraformaldehyde (PFA) solution, using the protocol outlined in our previous publication^12^. Briefly, after fixation, sections were washed with phosphate buffered saline (PBS) 3 times, and then stained with WGA (Sigma-Aldrich, St. Louis, MO) for 25 min, followed by another 3-time-wash in PBS. Sections were then mounted in ProLong Gold antifade reagent with DAPI (Thermo Fisher Scientific, Waltham, MA) and imaged under a fluorescent microscope. *ImageJ* software was used to quantify cells in sections at 20 × per each group (n = 3). Relative cell area was calculated for each group by dividing each total section area per counting region by the number of cells within the counted region.

### Statistical analysis

Multi-Way ANOVA was performed to estimate how the parameters studied were influenced by treatment, T1DM, and sex. Pairwise multiple comparisons using Tukey Honest significant difference (Tukey HSD) post-hoc testing was performed after a multi-factor ANOVA. All of these tests were run using R statistical program. The Pearson's correlation matrix of outcomes (such as body weight, heart weight, and so on) were also generated using the corrplot package in R.

## Supplementary Information

Supplementary Information.

## Abbreviations

CO: Cardiac output
CVD: Cardiovascular disease
EF: Ejection fraction
FS: Fractional shortening
H&E: Hematoxylin and eosin
HR: Heart rate
ICU: Intensive care unit
LVIDd: Left ventricular internal diameter end-diastolic
LVIDs: Left ventricular internal diameter end-systolic
Multi-factor ANOVA: Multi-way analysis of variance
SV: Stroke volume
T1DM: Type 1 diabetes mellitus
WGA: Wheat-germ agglutinin
