# Supplementary material for: The implications of hyperoxia, type 1 diabetes and sex on cardiovascular physiology in mice
Source: Sci Rep. 2021 Nov 29;11:23086. doi: 10.1038/s41598-021-02550-2 (PMC8630164; doi:10.1038/s41598-021-02550-2)
Supplement: Supplementary file 1 — Supplementary Information. [file 41598_2021_2550_MOESM1_ESM.pdf]

S1: Akita male normoxia

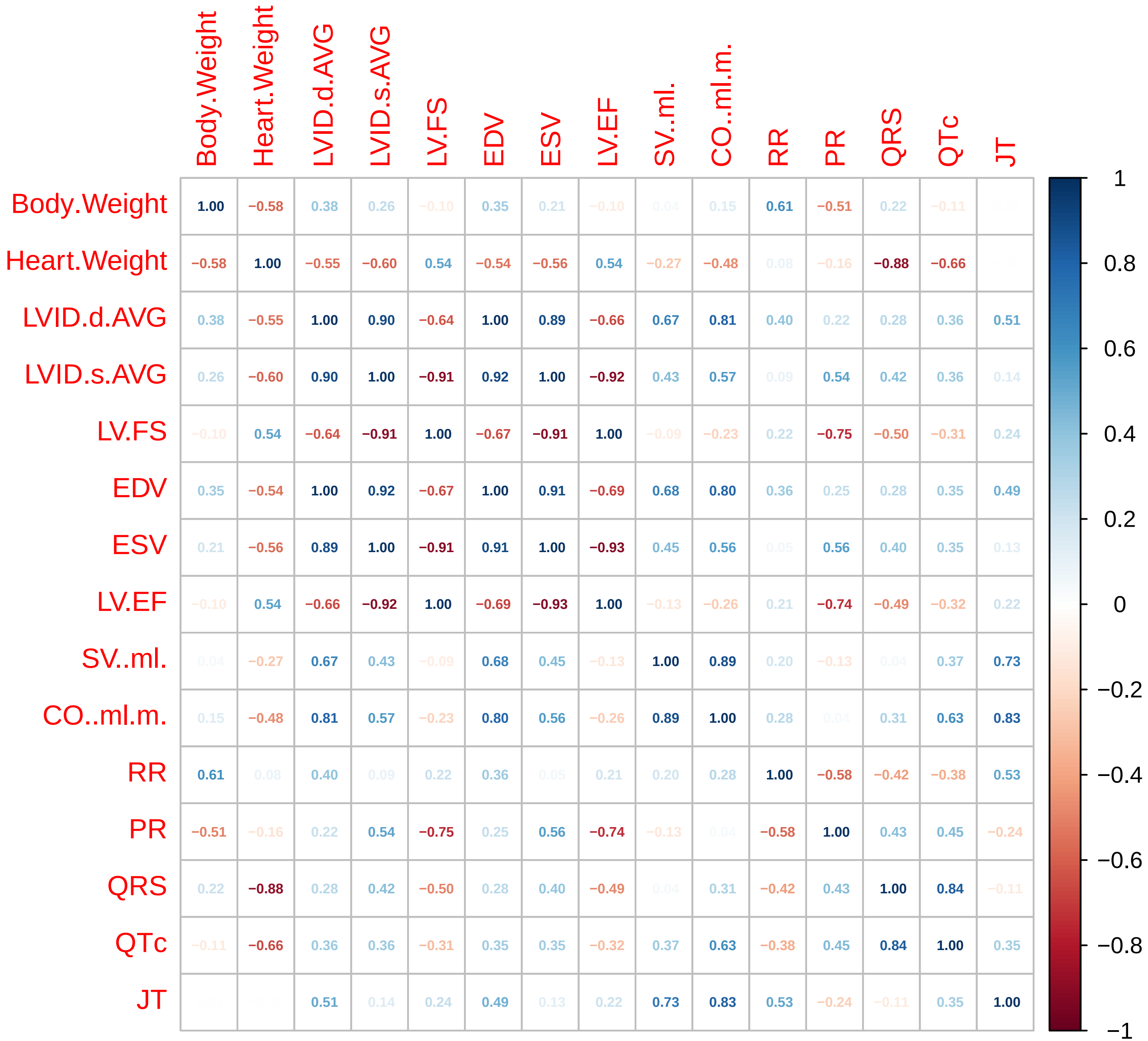

S2: Akita male hyperoxia

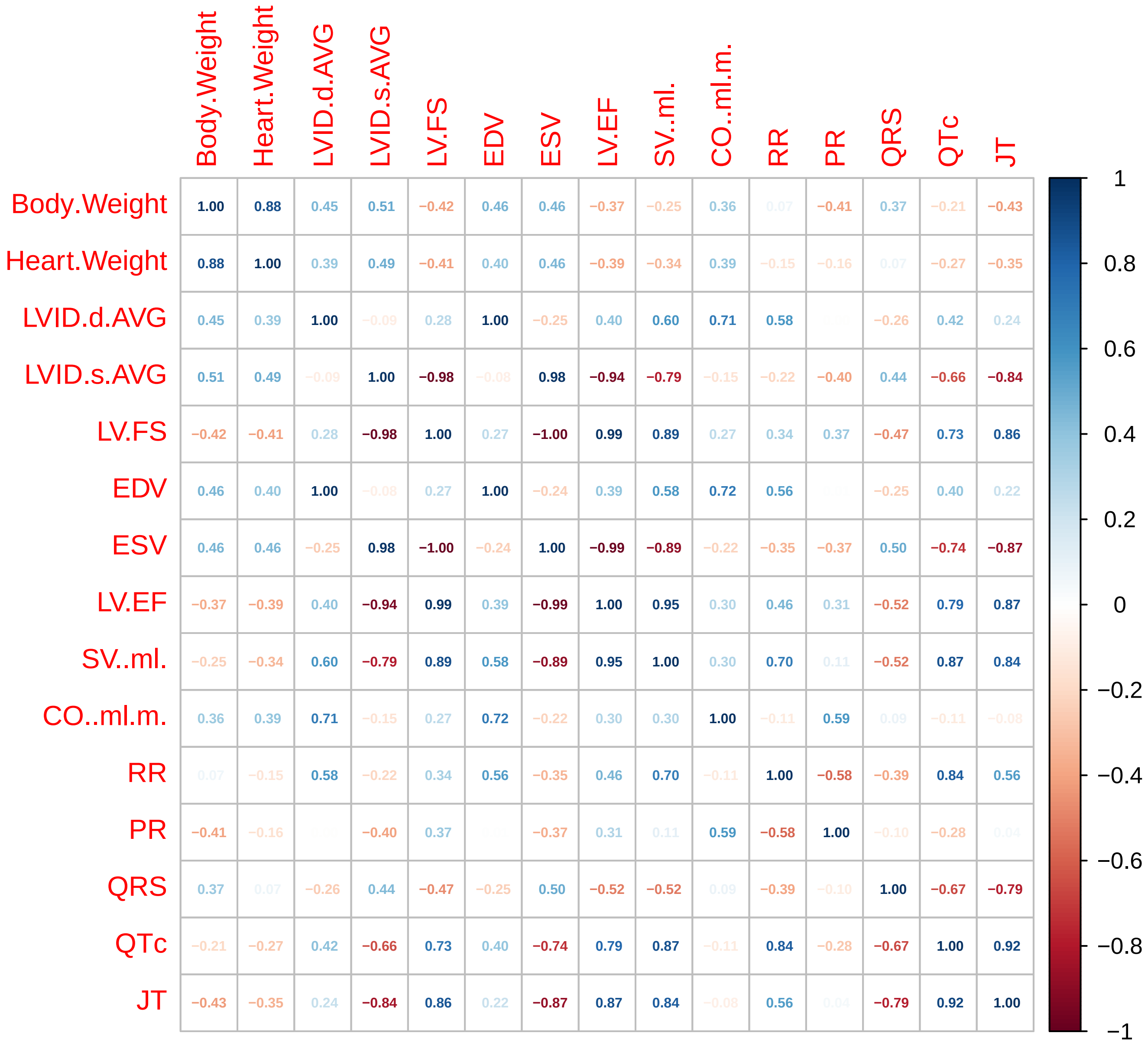

S3: Akita female normoxia

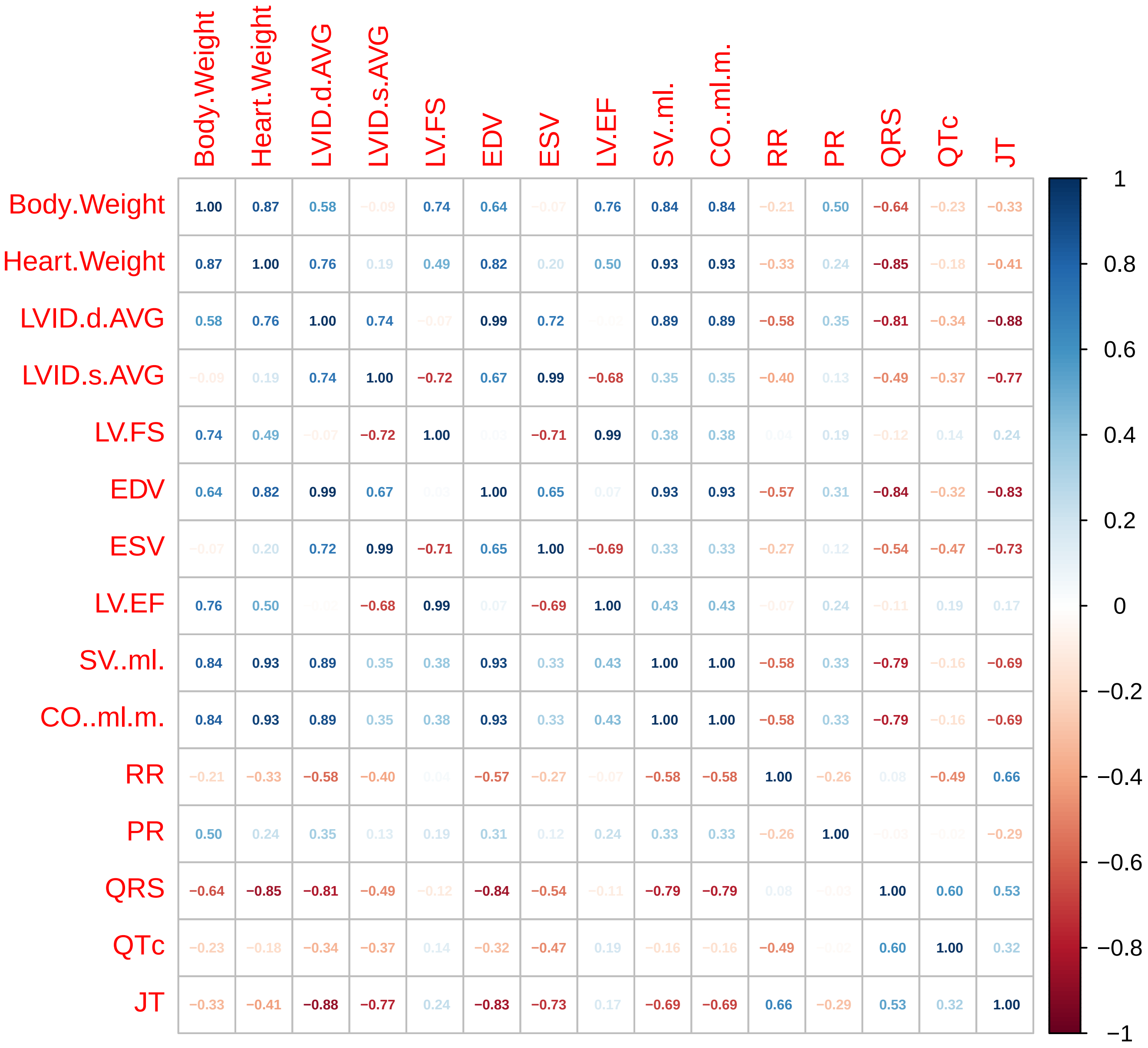

S4: Akita female hyperoxia

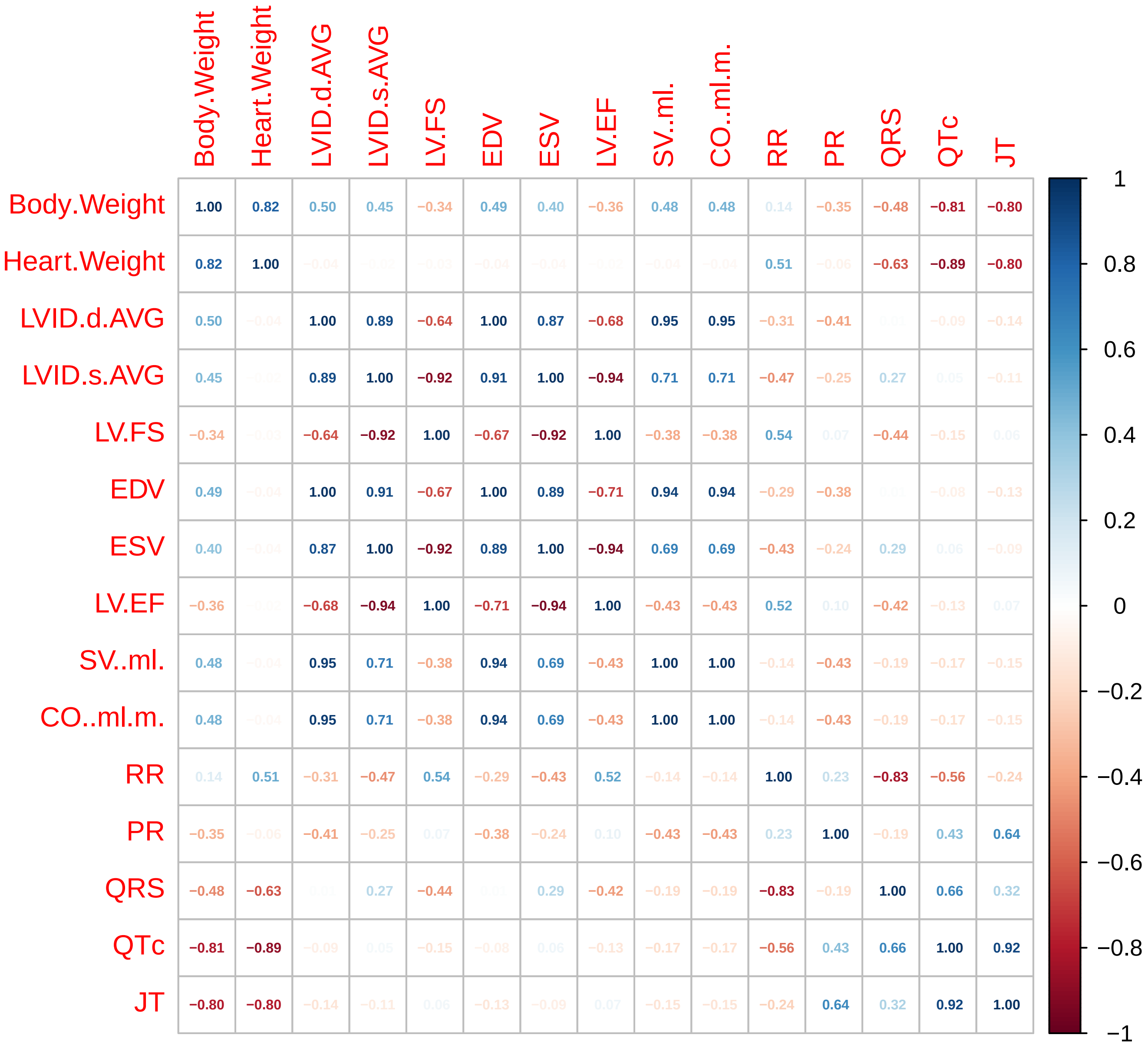

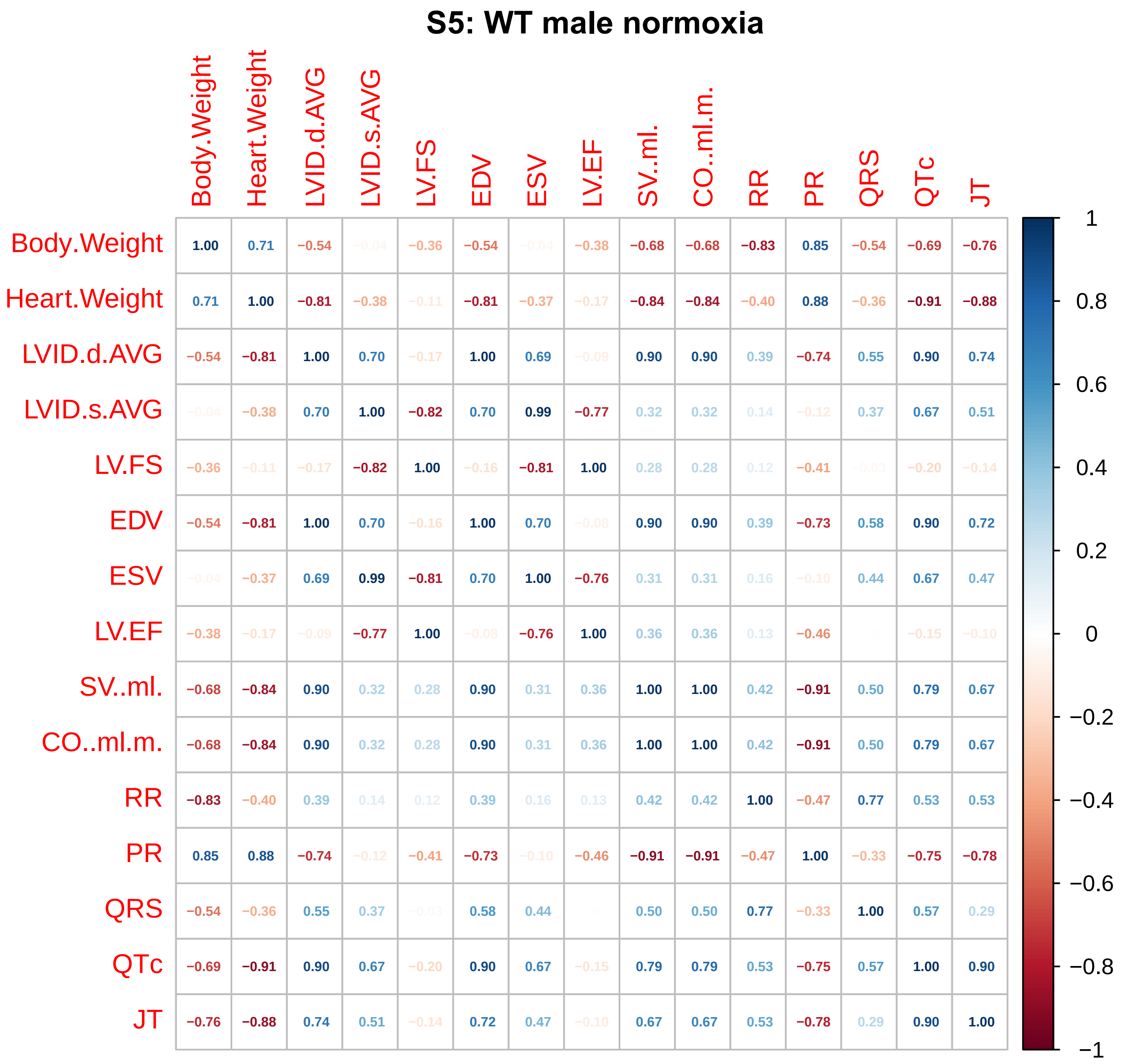
S5: WT male normoxia
Body.Weight Heart.Weight LVID.d.AVG LVID.s.AVG LV.FS EDV ESV LV.EF SV..ml. CO..ml.m. RR PR QRS QTc JT
Body.Weight 1.00 0.71 −0.54 −0.04 −0.36 −0.54 −0.04 −0.38 −0.68 −0.68 −0.83 0.85 −0.54 −0.69 −0.76
Heart.Weight 0.71 1.00 −0.81 −0.38 −0.11 −0.81 −0.37 −0.17 −0.84 −0.84 −0.40 0.88 −0.36 −0.91 −0.88
LVID.d.AVG −0.54 −0.81 1.00 0.70 −0.17 1.00 0.69 −0.09 0.90 0.90 0.39 −0.74 0.55 0.90 0.74
LVID.s.AVG −0.04 −0.38 0.70 1.00 −0.82 0.70 0.99 −0.77 0.32 0.32 0.14 −0.12 0.37 0.67 0.51
LV.FS −0.36 −0.11 −0.17 −0.82 1.00 −0.16 −0.81 1.00 0.28 0.28 0.12 −0.41 −0.03 −0.20 −0.14
EDV −0.54 −0.81 1.00 0.70 −0.16 1.00 0.70 −0.08 0.90 0.90 0.39 −0.73 0.58 0.90 0.72
ESV −0.04 −0.37 0.69 0.99 −0.81 0.70 1.00 −0.76 0.31 0.31 0.16 −0.10 0.44 0.67 0.47
LV.EF −0.38 −0.17 −0.09 −0.77 1.00 −0.08 −0.76 1.00 0.36 0.36 0.13 −0.46 −0.15 −0.10
SV..ml. −0.68 −0.84 0.90 0.32 0.28 0.90 0.31 0.36 1.00 1.00 0.42 −0.91 0.50 0.79 0.67
CO..ml.m. −0.68 −0.84 0.90 0.32 0.28 0.90 0.31 0.36 1.00 1.00 0.42 −0.91 0.50 0.79 0.67
RR −0.83 −0.40 0.39 0.14 0.12 0.39 0.16 0.13 0.42 0.42 1.00 −0.47 0.77 0.53 0.53
PR 0.85 0.88 −0.74 −0.12 −0.41 −0.73 −0.10 −0.46 −0.91 −0.91 −0.47 1.00 −0.33 −0.75 −0.78
QRS −0.54 −0.36 0.55 0.37 −0.03 0.58 0.44 0.50 0.50 0.77 −0.33 1.00 0.57 0.29
QTc −0.69 −0.91 0.90 0.67 −0.20 0.90 0.67 −0.15 0.79 0.79 0.53 −0.75 0.57 1.00 0.90
JT −0.76 −0.88 0.74 0.51 −0.14 0.72 0.47 −0.10 0.67 0.67 0.53 −0.78 0.29 0.90 1.00
1 0.8 0.6 0.4 0.2 0 −0.2 −0.4 −0.6 −0.8 −1

S6: WT male hyperoxia

	Body.Weight	Heart.Weight	LVID.d.AVG	LVID.s.AVG	LV.FS	EDV	ESV	LV.EF	SV..ml.	CO..ml.m.	RR	PR	QRS	QTc	JT
Body.Weight	1.00	0.97	−0.57	−0.72	0.68	−0.67	−0.88	0.81	−0.48	−0.50	−0.73	−0.55	−0.23	0.00	−0.50
Heart.Weight	0.97	1.00	−0.56	−0.74	0.74	−0.68	−0.90	0.85	−0.47	−0.52	−0.73	−0.52	−0.26	−0.06	−0.55
LVID.d.AVG	−0.57	−0.56	1.00	0.93	−0.72	0.98	0.84	−0.74	0.98	0.96	0.35	0.63	0.30	0.38	0.56
LVID.s.AVG	−0.72	−0.74	0.93	1.00	−0.91	0.97	0.95	−0.93	0.89	0.90	0.45	0.68	0.30	0.41	0.69
LV.FS	0.68	0.74	−0.72	−0.91	1.00	−0.79	−0.88	0.98	−0.65	−0.69	−0.38	−0.62	−0.17	−0.45	−0.72
EDV	−0.67	−0.68	0.98	0.97	−0.79	1.00	0.92	−0.82	0.97	0.97	0.48	0.70	0.40	0.38	0.64
ESV	−0.88	−0.90	0.84	0.95	−0.88	0.92	1.00	−0.95	0.78	0.82	0.65	0.67	0.37	0.27	0.68
LV.EF	0.81	0.85	−0.74	−0.93	0.98	−0.82	−0.95	1.00	−0.66	−0.70	−0.52	−0.61	−0.22	−0.35	−0.71
SV..ml.	−0.48	−0.47	0.98	0.89	−0.65	0.97	0.78	−0.66	1.00	0.98	0.32	0.66	0.39	0.42	0.55
CO..ml.m.	−0.50	−0.52	0.96	0.90	−0.69	0.97	0.82	−0.70	0.98	1.00	0.41	0.67	0.50	0.41	0.59
RR	−0.73	−0.73	0.35	0.45	−0.38	0.48	0.65	−0.52	0.32	0.41	1.00	0.16	0.71	−0.39	0.18
PR	−0.55	−0.52	0.63	0.68	−0.62	0.70	0.67	−0.61	0.66	0.67	0.16	1.00	0.16	0.67	0.77
QRS	−0.23	−0.26	0.30	0.30	−0.17	0.40	0.37	−0.22	0.39	0.50	0.71	0.16	1.00	−0.29	−0.01
QTc	0.00	−0.06	0.38	0.41	−0.45	0.38	0.27	−0.35	0.42	0.41	−0.39	0.67	−0.29	1.00	0.82
JT	−0.50	−0.55	0.56	0.69	−0.72	0.64	0.68	−0.71	0.55	0.59	0.18	0.77	−0.01	0.82	1.00

1 0.8 0.6 0.4 0.2 0 −0.2 −0.4 −0.6 −0.8 −1

S7: WT female normoxia

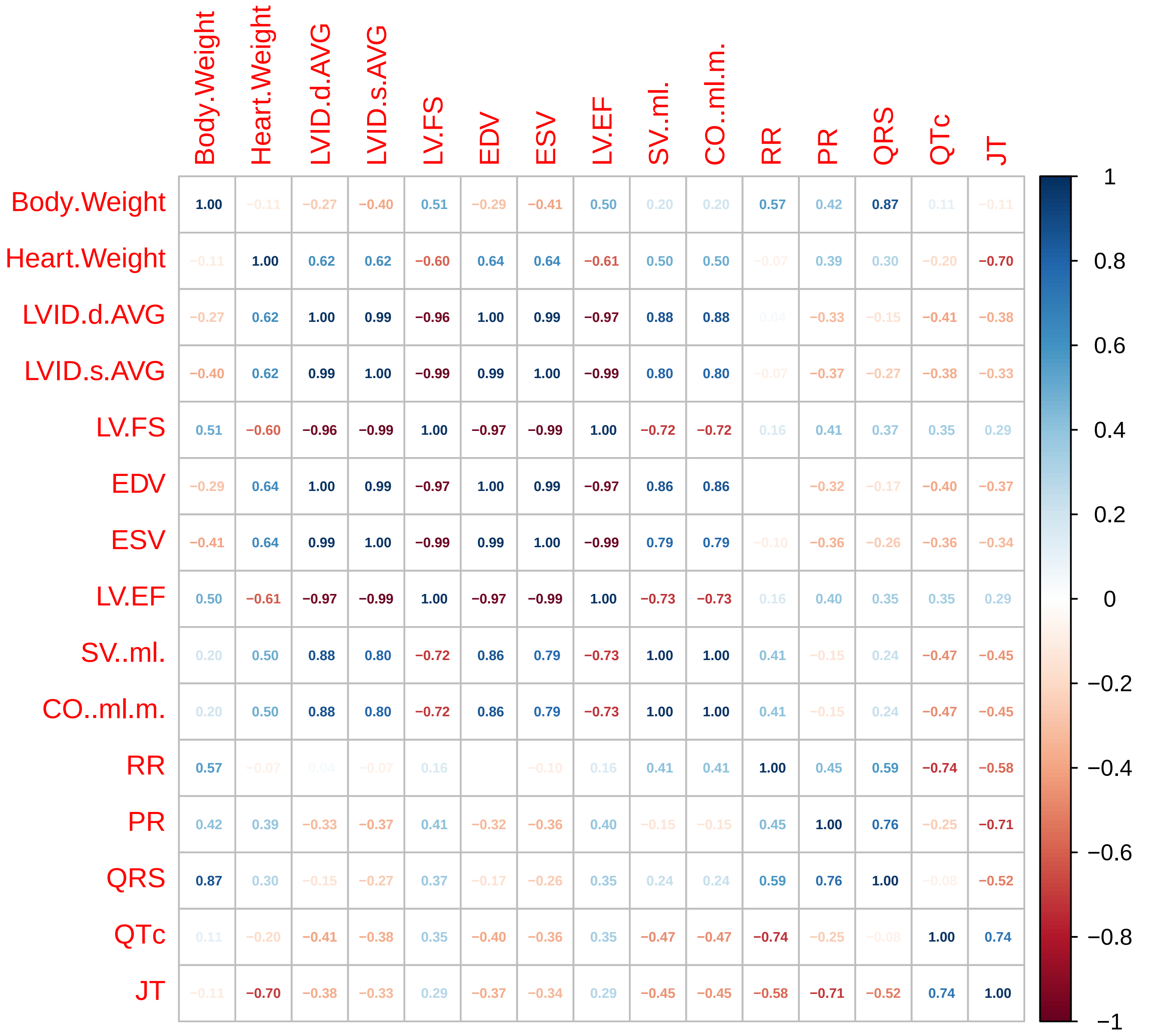

S8: WT female hyperoxia

	Body.Weight	Heart.Weight	LVID.d.AVG	LVID.s.AVG	LV.FS	EDV	ESV	LV.EF	SV..ml.	CO..ml.m.	RR	PR	QRS	QTc	JT
Body.Weight	1.00	0.82	−0.78	−0.75	0.70	−0.79	−0.73	0.66	−0.71	−0.71	0.32	−0.27	−0.66	−0.16	0.45
Heart.Weight	0.82	1.00	−0.33	−0.32	0.29	−0.35	−0.33	0.27	−0.31	−0.31	−0.22	−0.35	−0.95	0.28	0.65
LVID.d.AVG	−0.78	−0.33	1.00	0.95	−0.89	1.00	0.90	−0.84	0.92	0.92	−0.77	0.27	0.15	0.44	−0.28
LVID.s.AVG	−0.75	−0.32	0.95	1.00	−0.99	0.96	0.99	−0.96	0.76	0.76	−0.85	0.02	0.20	0.61	−0.16
LV.FS	0.70	0.29	−0.89	−0.99	1.00	−0.90	−1.00	1.00	−0.64	−0.64	0.87	0.11	−0.21	−0.69	0.08
EDV	−0.79	−0.35	1.00	0.96	−0.90	1.00	0.91	−0.85	0.91	0.91	−0.77	0.26	0.18	0.44	−0.29
ESV	−0.73	−0.33	0.90	0.99	−1.00	0.91	1.00	−0.99	0.66	0.66	−0.85	−0.09	0.24	0.67	−0.11
LV.EF	0.66	0.27	−0.84	−0.96	1.00	−0.85	−0.99	1.00	−0.56	−0.56	0.86	0.20	−0.21	−0.74	0.02
SV..ml.	−0.71	−0.31	0.92	0.76	−0.64	0.91	0.66	−0.56	1.00	1.00	−0.56	0.55	0.09	0.14	−0.42
CO..ml.m.	−0.71	−0.31	0.92	0.76	−0.64	0.91	0.66	−0.56	1.00	1.00	−0.56	0.55	0.09	0.14	−0.42
RR	0.32	−0.22	−0.77	−0.85	0.87	−0.77	−0.85	0.86	−0.56	−0.56	1.00	0.27	0.31	−0.85	−0.27
PR	−0.27	−0.35	0.27	0.02	0.11	0.26	−0.09	0.20	0.55	0.55	0.27	1.00	0.29	−0.73	−0.86
QRS	−0.66	−0.95	0.15	0.20	−0.21	0.18	0.24	−0.21	0.09	0.09	0.31	0.29	1.00	−0.34	−0.69
QTc	−0.16	0.28	0.44	0.61	−0.69	0.44	0.67	−0.74	0.14	0.14	−0.85	−0.73	−0.34	1.00	0.66
JT	0.45	0.65	−0.28	−0.16	0.08	−0.29	−0.11	0.02	−0.42	−0.42	−0.27	−0.86	−0.69	0.66	1.00

Color scale: 1, 0.8, 0.6, 0.4, 0.2, 0, −0.2, −0.4, −0.6, −0.8, −1
